# Beyond antimicrobial resistance: MATE-type efflux pump FepA contributes to flagellum formation and virulence in *Listeria monocytogenes*

**DOI:** 10.1128/aem.00462-25

**Published:** 2025-06-10

**Authors:** Jing Xia, Guo Jiang, Yaru Luo, Zhe Wang, Jingjing Li, Zhanhong Fu, Qian Qin, Jiali Xu, Simin Deng, Mianmian Chen, Yue Han, Lingli Jiang, Houhui Song, Changyong Cheng

**Affiliations:** 1 Key Laboratory of Applied Biotechnology on Animal Science & Veterinary Medicine of Zhejiang Province, Zhejiang Engineering Research Center for Veterinary Diagnostics & Advanced Technology, Zhejiang International Science and Technology Cooperation Base for Veterinary Medicine and Health Management, Belt and Road International Joint Laboratory for One Health and Food Safety, China-Australia Joint Laboratory for Animal Health Big Data Analytics, College of Veterinary Medicine of Zhejiang A&F University, Hangzhou, Zhejiang, China; 2 Ningbo College of Health Sciences 200642, Ningbo, Zhejiang, China; Anses, Maisons-Alfort Laboratory for Food Safety, Maisons-Alfort, France

**Keywords:** MATE-type efflux pump, FepA, antimicrobial resistance, flagellum formation, virulence, *Listeria monocytogenes*

## Abstract

*Listeria monocytogenes* is commonly found in nature and can readily contaminate various food products. Efflux pump proteins represent an essential group of proteins in bacteria, playing key roles in numerous biological processes. This study investigates the contribution of FepA to motility and virulence apart from antimicrobial resistance in *L. monocytogenes*. The minimum inhibitory concentrations of various antimicrobials and the survival of *L. monocytogenes* in medium containing these agents were assessed. Loss of FepA increased sensitivity to a range of antimicrobial agents and significantly impaired growth under antimicrobial pressure. We examined bacterial flagellum formation, flagellar gene transcription, and protein expression. Results indicated a marked decrease in flagellum formation in Δ*fepA* mutants, owing to reduced expression of key flagellar proteins such as FlhF and FlgG. In addition, results from cell infection, virulence genes transcription, and protein expression experiments revealed that FepA deletion diminished bacterial invasiveness and intracellular proliferation, correlating with decreased secretion of virulence proteins, including InlB, InlC, Mpl, PlcA, and LLO. These findings indicate that FepA is integral not only to antimicrobial resistance and *in vitro* adaptability but also to flagellar formation and virulence. This research helps deepen the understanding of mechanisms underlying drug resistance and pathogenicity in the significant foodborne pathogen *L. monocytogenes*.

**IMPORTANCE:**

*Listeria monocytogenes* is a significant zoonotic foodborne intracellular pathogen with a mortality rate of up to 20%–30%. This bacterium employs various mechanisms, including efflux pumps, to enhance its environmental adaptability and maintain infectivity. In this study, we discovered that the MATE-type multidrug efflux pump protein FepA is not only associated with bacterial resistance to multiple antimicrobials but also plays a crucial role in promoting flagellum formation, which is essential for motility and resistance to adverse environmental conditions. Additionally, FepA is involved in the secretion of virulence proteins, facilitating bacterial invasion and proliferation within the host. Our findings reveal, for the first time, that the multidrug efflux pump FepA contributes to flagellar formation and virulence, providing new insights into the mechanisms of environmental adaptation and virulence expression in *L. monocytogenes* and aiding in the discovery of potential therapeutic targets.

## INTRODUCTION

*Listeria monocytogenes* is a principal zoonotic foodborne intracellular pathogen, which is commonly found in nature. *Listeria monocytogenes* can accommodate different environments (such as the range of 4–45°C, hyperosmolar environment, broad pH range, etc.) and can survive in various food items (including meat and its derivatives, dairy products, vegetables, fruits, fish, etc.) and processing environments (1–3). Thus, consumption of food tainted with *L. monocytogenes* could lead to a serious invasive infection called listeriosis in humans and animals (2, 3). *Listeria* infection can be especially harmful for immunocompromised people, the elderly, and pregnant women and may cause severe symptoms like sepsis, abortion, and meningitis (4). Being one of the food-borne pathogens with the highest fatality rate (20–30%), the FDA and USDA take a zero-tolerance policy for *L. monocytogenes* in ready-to-eat (RTE) foods (5, 6). Over the last 20 years, many outbreaks due to active surveillance for listeriosis have been reported in the United States and European countries, and the vast majority of listeriosis cases were caused by consumption of contaminated RTE foods (7, 8). In recent years, the epidemiological investigation witnessed *L. monocytogenes* contamination existing in different food processing processes in different provinces and cities in China (9, 10), raising the risk of getting infection.

Effective antibiotic therapy (e.g., the combination of ampicillin and gentamicin) is currently the only viable option for *L. monocytogenes* infections. Generally, *L. monocytogenes* is often sensitive to multiple drugs, but as a result of the selective pressure from the excessive use of antimicrobials, antibiotic resistance, including multidrug resistance (MDR) *L. monocytogenes* is increasingly reported (11–13). According to previous reports, the mechanisms of antimicrobial resistance in *L. monocytogenes* are rather intricate, including resistance genes transfer mediated by mobile elements, efflux pumps, production of passivating enzymes or inactivated enzymes, biofilms, and other mechanisms (11, 14–16). As one of the main mechanisms by which *L. monocytogenes* develops MDR, efflux pumps provide resistance to various antimicrobials, such as antibiotics, disinfectants, heavy metals, and other antibacterial or toxic substances. In the past three decades, multiple efflux pumps have been validated within *L. monocytogenes*, which are closely tied with the pump to a variety of substances, and some efflux pumps are also associated with other functions, such as adaptation to adverse environments and pathogenicity (15, 17–19).

CadA was the initial efflux pump found in *L. monocytogenes*, exhibiting resistance to cadmium first shown to be plasmid-borne (20), and further studies found that the CadA operon located on the Tn*5422* transposon was a key gene mediating cadmium tolerance in *L. monocytogenes* (17). Depending on the sequence differences, four CadAs have been described in *L. monocytogenes*, namely CadA1–CadA4 (17, 21–23). CadA mediates Cd^2+^ efflux, and CadC is a negative regulatory protein (24). In addition to regulating *cadA*, *cadC* has also been shown to be a potential suppressor of a wide range of virulence gene expression. Pombinho et al. (25) showed that CadC was significantly upregulated during the advanced phase of *L. monocytogenes* infection, leading to the suppression of genes necessary for gastrointestinal tract survival to promote bacterial virulence and thus infection. Studies using the *Galleria mellonella* model revealed that an active CadA4 decreased virulence, possibly facilitating the establishment of commensal colonization in the insect larvae. Experiments assessing biofilm formation indicated that disabling *cadA4* lowered the formation of biofilm (26). Another ATP-binding cassette (ABC) superfamily efflux pump, *virAB* in *L. monocytogenes,* confers resistance to cephalosporins, nisin, ethidium bromide (EtBr), and some other antimicrobial agents (18). The VirAB-deficient strain exhibited similar defects in the formation of plaque *in vitro* and virulence *in vivo*, characterized by shorter actin tails during intracellular infection, indicating a limited capacity to move and disseminate through actin-based motility (27). Both VirAB and the regulator VirS play crucial roles in biofilm formation, potentially working together in the process within *L. monocytogenes*. The findings further indicate that VirAB and VirS are implicated in attachment but not linked to swarming motility (16).

In addition, the MDR MFS-type efflux pumps, MdrT and MdrM, can activate the cytoplasmic monitoring pathway of the host innate immunity by secreting c-di-AMP, cause the release of IFN-β, and promote the infection of *L. monocytogenes* (28). Other than MdrT and MdrM, the homologous proteins MdrA and MdrC of MdrM (comprising the MdrMTAC complex) collectively participate in the induction and release of type I interferon. Their transcription is upregulated during intracellular growth and promotes the host cell response to type I interferon, indicating a close association between MdrT/MdrM and bacterial virulence expression. The mutant lacking four transporter genes (Δ*mdrMTAC*) displayed sensitivity to sublethal doses of vancomycin because it could not generate and release peptidoglycan under such conditions (29). Taken together, the findings indicate a physiological connection exists between c-di-AMP and the MDR transporters, supporting the hypothesis that MDR transporters facilitate c-di-AMP secretion to govern peptidoglycan synthesis in response to cell wall stress (29). However, there are many other suspected multidrug-resistant efflux pumps in *L. monocytogenes*, and their related functions have not been discovered and reported. It is also unknown whether there are other efflux pumps associated with virulence in *L. monocytogenes*.

Previously, reports indicated that in *L. monocytogenes* BM4716, a single point mutation of the TetR-type transcriptional regulatory gene *fepR* was introduced (30). The framing shift results in the introduction of early stop codons, which become inactive truncated proteins that are located in FepR. The downstream MATE-type (the multidrug and toxic compound extrusion family) efflux pump is overexpressed, thus mediating resistance to ciprofloxacin, norfloxacin, and EtBr (30). Upon the sequence analysis of *L. monocytogenes* EGD-e, we found that the *lmo2087* gene was also a FepA-like efflux pump gene, and its protein sequence was 98% similar to that of FepA in BM4716, with only eight amino acids different, so it was also named FepA. The high similarity of sequences suggests that the function should be similar. However, more than antimicrobial resistance, we found that FepA contributes to flagellum formation and virulence, which provide more useful data to the research of efflux pumps in *L. monocytogenes*.

## MATERIALS AND METHODS

### Bacterial strains, plasmids, primers, and culture conditions

The wild-type strain used was *L. monocytogenes* EGD-e. *Listeria monocytogenes* EGD-e is originated from EGD (31). The EGD-e genome sequence is well-defined and serves as a common *L. monocytogenes* standard strain for researching environmental adaptation and virulence of *L. monocytogenes* (2, 32–34). Plasmids pKSV7 and pIMK2 stocked in *Escherichia coli* (*E. coli*) DH5α were utilized to construct gene deletion and complement strains, respectively. *Listeria* and *E. coli* strains were cultured in brain-heart infusion (BHI) and Luria-Bertani (LB) medium (Oxoid), respectively. Ampicillin (50 µg/mL), chloramphenicol (10 µg/mL), or kanamycin (50 µg/mL) was supplemented to the media at the specified final concentrations as needed. All chemicals used were sourced from Sigma-Aldrich, Merck, or Sangon Biotech (Shanghai, China) and were of high purity. The primers employed in this study are detailed in Table S1.

### Construction of FepA deletion and complement strains

A previously used homologous recombination approach utilizing the splicing by overlap extension PCR technique was employed for creating in-frame gene deletion strains (35). Briefly, gene deletion was introduced into the *L. monocytogenes* strain EGD-e employing the temperature-sensitive shuttle vector pKSV7. The recombinant plasmid that contains the deletion cassette of the target gene was then introduced into *E. coli* DH5α. Following sequence verification, the recombinant vector was introduced into competent *L. monocytogenes* cells via electroporation. Selected transformants were subsequently serially passaged at 42°C to facilitate chromosomal integration, followed by 30°C to induce excision and removal of the plasmid. The resulting colonies sensitive to chloramphenicol were identified as recombinants, and the gene deletion was qualified through PCR and DNA sequencing, denoted as Δ*fepA*.

To establish the Δ*fepA* complement strain, we used the integrative plasmid pIMK2 (kanamycin resistant). The full open reading frame (ORF) of *fepA*, along with its promoter region, was amplified and inserted into pIMK2 after digestion with the appropriate restriction enzymes to eliminate the *P*help region. The resultant plasmids were then electroporated into the *L. monocytogenes* Δ*fepA* strain. The complement strain was confirmed by antibiotic resistance, PCR, and DNA sequencing, named as CΔ*fepA*.

### Bacterial morphology and minimum inhibitory concentration (MIC) assays

The wild-type *L. monocytogenes* and gene deletion mutants were incubated on BHI plates for a duration of 12 hours, followed by observing the morphology of bacterial colonies utilizing a stereomicroscope. MICs of various antibiotics were assessed using the broth microdilution method, following the guidelines set by the Clinical and Laboratory Standards Institute in the United States (36). MICs of antiseptics and dyes were also determined by the broth dilution method (37). The antimicrobials tested included ampicillin (AMP), cefotaxime (CTX), chloramphenicol (CHL), kanamycin (KAN), gentamicin (GEN), bacitracin (BAC), erythromycin (ERY), clindamycin (CLI), tetracycline (TET), ciprofloxacin (CIP), nisin, benzalkonium bromide (BB), benzalkonium chloride (BC), triclosan, chlorhexidine (Chlo), EtBr, and acriflavine (AF). Three colonies from each strain were selected from Mueller-Hinton plates, transferred into 9 mL of cation-adjusted Mueller-Hinton broth (CAMHB), and then cultivated at 37°C for 18–24 hours. This method is performed in a 96-well plate with a total volume of 200 µL. Prepare serially double-diluted antibiotic solutions in sterile CAMHB and add an equal volume of the diluted bacterial suspension (10^5^ CFU/mL). The MIC was defined as the lowest concentration of the antimicrobial agent required to hinder bacterial growth following an incubation period of 24 hours at 37°C. *E. coli* ATCC 25922 and *S. aureus* ATCC 25923 served as quality controls. Attention was mainly given to MIC alterations due to gene deletion. Reserpine (10 µg/mL) was added to antimicrobials with modified MIC values to further observe the changes (38). Three standalone experiments were used to determine MICs.

### Growth or survival ability of *L. monocytogenes* in medium with or without antimicrobial agents

*Listeria monocytogenes* strains (EGD-e, Δ*fepA*, and CΔ*fepA*) were cultured overnight, harvested via centrifugation at 5,000 × *g* at 4°C, rinsed twice in phosphate buffered saline (PBS, 10 mM, pH 7.4), and standardized to an OD_600 nm_ of 0.6 using PBS. Bacteria were diluted 1:100 in blank BHI broth or BHI with different concentrations of antimicrobial agents (4, 8 µg/mL CTX; 1, 2 µg/mL BB; 2, 4 µg/mL EtBr) based on the MIC results above. Bacterial growth at OD_600 nm_ was monitored kinetically at 1 hour intervals during a 12 hour incubation period. Growth data in liquid medium are presented with a logarithmic *Y*-axis. Additionally, the growth rates during the exponential phase for three independent cultures per strain, along with statistical analysis for comparison, are calculated using GraphPad Prism 9.0. Statistical analysis of the apparently restricted growth curves involved examining both the growth rate and lag time. Simultaneously, bacteria cultured in blank BHI broth for 6 hours underwent 10-fold serial dilution, and 10 µL of each dilution was then placed onto BHI plates with the respective antimicrobial concentrations. Plates were incubated overnight at 37°C to observe the survival ability of the three strains further. Growth analysis at 30°C in BHI broth and on plates followed the same procedure. Three independent experiments have been conducted in this portion.

### Motility assay and observation of bacterial flagellum morphology

The motility assay and observation of bacterial flagellum morphology were conducted as previously outlined (39). Overnight-cultured *L. monocytogenes* strains were harvested and standardized to an OD_600 nm_ of 0.20 (2 × 10^8^ CFU/mL). Subsequently, 5 µL of bacterial samples were inoculated onto the 0.25% TSA plates and cultured at 30°C and 37°C, respectively, for 48 hours. Motility was evaluated by observing the outward movement of bacteria through the agar from the inoculation point. Data are expressed as mean ± SD of three independent experiments. For flagellum morphology observation, colonies grown overnight on BHI plates at 30°C were resuspended in monoethanolamine buffer (50 µL, pH 10.0). A 10 µL of the suspension was placed onto carbon-coated copper grids and allowed to incubate for 2 minutes. The bacteria were dyed with 0.5% phosphotungstic acid (pH 7.0), and the grids were allowed to air-dry and analyzed with a transmission electron microscope (Hitachi High-Technologies Corporation, Tokyo, Japan).

### Adhesion, invasion, and proliferation in Caco-2 cells

Overnight-cultured *L. monocytogenes* strains were rinsed and suspended in PBS (10 mM, pH 7.4). Caco-2 cell monolayers, maintained in RPMI1640 supplemented with 20% fetal bovine serum (FBS) from HyClone in Chicago, IL, USA, were exposed to the bacteria at a multiplicity of infection (MOI) of 10:1 for 30 minutes. Following three rounds of washing with PBS, cells were subjected to lysis for adhesion studies. For invasion assessments, cells were exposed to bacteria for a duration of 90 minutes, treated with 50 µg/mL of gentamicin in RPMI1640 for another 90 minutes to eliminate extracellular bacteria, followed by lysis using 1 mL of chilled sterile water. Lysates were serially 10-fold diluted to quantify viable bacteria on BHI plates. Adhesion was calculated by evaluating the ratio of recovered colonies to the initially inoculated colonies, while invasion was determined as the ratio of colonies retrieved post-gentamicin treatment to inoculated colonies. Furthermore, infected cells were cultured for six more hours, lysed, and intracellular bacteria were enumerated using the same method. The number of bacteria able to invade cells and survive is expressed as mean ± SD of the recovery rate for each strain (three replicates).

### Proliferation in RAW264.7 macrophages

The evaluation of bacterial proliferation ability in RAW264.7 macrophages refers to previous experimental methods (40). RAW264.7 cell monolayers were maintained in Dulbecco's modified eagle medium (DMEM) supplemented with 10% FBS. In brief, RAW264.7 cell monolayers were exposed to the stationary phase of *L. monocytogenes* strains at an MOI of 1:4 for 30 minutes. Following this, the cells underwent two washes with pre-warmed PBS prior to media replacement, and gentamicin (50 µg/mL) was added 1 hour post-infection to eliminate any extracellular bacteria. Cells were lysed at 0.5, 2, 5, or 8 hours post-infection by introducing 1 mL of chilled sterile water, and the resulting lysates were diluted 10 times for counting viable bacteria on BHI agar plates. The number of recovered bacteria able to invade cells and survive is expressed as mean ± SD of three replicates for each strain.

### Plaque-forming assay on mouse fibroblast L929 cells

The plaque assay procedure was conducted using mouse fibroblast L929 cells based on a previous report (40). Briefly, L929 cell monolayers were maintained in DMEM supplemented with 10% FBS and 2 mM L-glutamine. Cells, with a density of about 1 × 10^6^ cells per well, were infected at an MOI of 1:5 with *L. monocytogenes* for 1 hour (at 37°C with 5% CO_2_). To remove extracellular bacteria, gentamicin (50 µg/mL) was applied. Afterward, the cells were covered with 3 mL of medium containing agarose (0.7%) and gentamicin (10 µg/mL). Following a 72 hour incubation, the cells were fixed with 4% paraformaldehyde in PBS for 20 minutes and subsequently stained with crystal violet for plaque visualization. The dimensions and number of plaques generated by the mutant strains were calculated as a percentage of the plaques from the wild-type strain, assigning a plaque size of 100% to the wild-type EGD-e strain. This experiment has been repeated individually three times.

### Transcriptional change analysis of flagellum-associated genes and virulence genes

Quantitative reverse transcription PCR (RT-qPCR) was employed for analyzing transcriptional change analysis of flagellum-associated genes and virulence genes. The *L. monocytogene*s EGD-e and Δ*fepA* were grown until reaching stationary phase (OD_600 nm_ of 0.6) in BHI broth without agitation at 30°C. The bacteria were collected through centrifugation, and total RNA was extracted via the Trizol method. Genomic DNA was eliminated using DNase I (TaKara), and RNA purity was evaluated with the NanoDrop (Thermo Fisher Scientific) (35). Subsequently, RT-qPCR was conducted using the SYBR qPCR mix from TOYOBO to quantify the expression levels of various flagellum-associated genes including *fliD*, *flgB*, *flgK*, *fliR*, *fliG*, *flgD*, *fliP*, *flhB*, *fliM*, *motB*, *gmaR*, *flaA*, *mogR*, *flhA*, *flgG*, *flgE*, *flgC*, *flgL*, *fliF*, *fliE*, *flil*, *fliH*, *fliQ*, *flhF,* and *fliY*. Normalization was achieved using the housekeeping gene 16S rRNA as an internal control. This analysis was performed with specific primer pairs using the LightCycler 480 Instrument II from Roche Diagnostics, USA. The relative transcription levels were calculated following the 2^−ΔΔCT^ strategy and presented as fold changes in relation to the control measurements (41). Each transcriptional analysis was conducted three times keeping the experimental conditions consistent. The transcriptional level analysis of virulence genes (*prfA*, *hly*, *mpL*, *plcA*, *plcB*, *actA*, *inlA*, *inlB,* and *inlC*) was the same as the above method, except that the strains were cultured at 37°C with shaking. Data are expressed as mean ± SD for three replicates.

### Analysis of expression levels of flagellin and virulence proteins

To explore the potential role of FepA in bacterial flagellar synthesis and expression of virulence proteins, western blot (WB) was utilized to examine the expression changes of flagellar-associated and virulence proteins. Polyclonal antibodies used here were prepared and stored in our laboratory previously, which were obtained by injecting New Zealand white rabbits (39). Overnight cultures of *L. monocytogenes* wild-type EGD-e, Δ*fepA,* and CΔ*fepA* were diluted in 100 mL of sterile BHI broth and cultivated until reaching the stationary phase at either 30°C or 37°C. Bacterial pellets and culture supernatants were harvested to isolate the various cell fractions. Secreted proteins were isolated from the culture supernatants. After centrifugation and filtration of the culture supernatant, trichloroacetic acid was added to precipitate the proteins, which were then washed and resuspended in SDS-PAGE sample buffer. The samples were boiled, stored at −20°C, and subsequently subjected to 12% SDS-PAGE. Immunoblotting was performed using antibodies against various flagellum-associated proteins (MogR, GmaR, FliY, FliM, FlhF, and FlgG) and virulence proteins (InlA, InlB, InlC, Mpl, PlcA, PlcB, LLO, and ActA). Glyceraldehyde 3-phosphate dehydrogenase and P60 served as internal controls, respectively. The WB results of repeated experiments were analyzed by using ImageJ software for quantitation.

### Statistical analysis

The experiments were replicated three times, and the corresponding data were assessed using the unpaired two-tailed Student’s *t*-test. Differences that were defined as statistically significant had a *P*-value < 0.05.

## RESULTS

### FepA is unnecessary for growth and colony morphology of *L. monocytogenes in vitro* at 37°C

**Fig 1:**
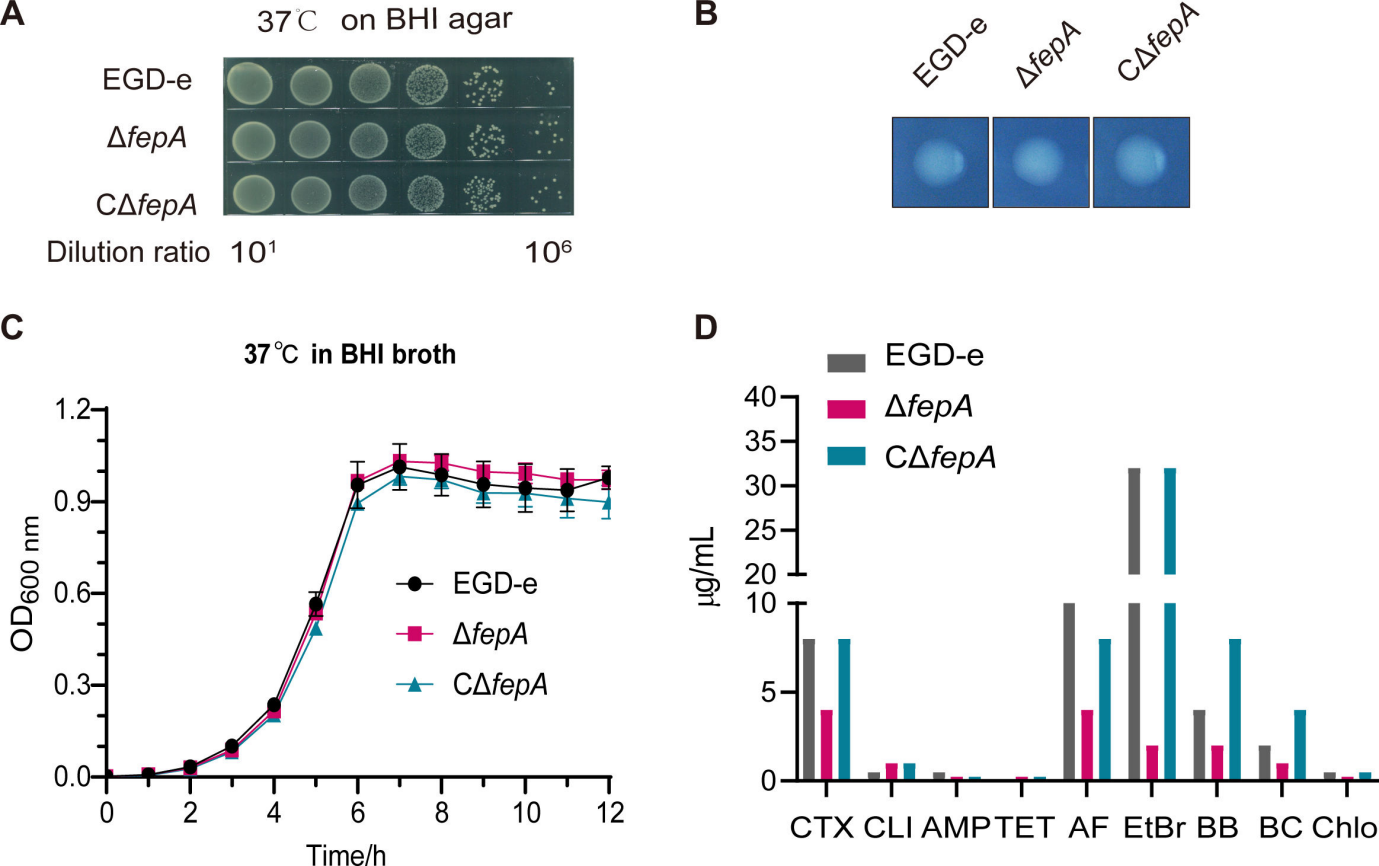
Loss of FepA increased sensitivity to a variety of antimicrobial agents in *L. monocytogenes*. Plate culture (**A**), bacterial colony morphology (**B**), and growth curves (**C**) of wild-type EGD-e and *fepA* mutants at 37°C. (**D**) MIC results to the antimicrobial agents of wild-type EGD-e and *fepA* mutants. CTX, cefotaxime; CLI, clindamycin; AMP, ampicillin; TET, tetracycline; AF, acriflavine; EtBr, ethidium bromide; BB, benzalkonium bromide; BC, benzalkonium chloride; Chlo, chlorhexidine. MIC values without or with 10 µg/mL reserpine are presented in Table S2. (**A and B**) Representative images of three independent experiments are shown. (**C**) The growth curves are shown with a logarithmic *Y*-axis. Data are expressed as mean ± SD for three replicates. Growth rates in the exponential phase of three independent cultures per strain plus statistical analysis for comparison are calculated using GraphPad Prism 9.0. ns, no significance.

As shown in Fig. 1C, the growth curves of Δ*fepA* and CΔ*fepA* were similar to the wild-type strain EGD-e (*P* = 0.5142 and *P* = 0.6130, respectively). Plate culture findings following 6 hours of growth in liquid culture indicated that the growth potential of *L. monocytogenes in vitro* remained unaffected in the absence of FepA (Fig. 1A). This finding suggests that the loss of FepA does not impact the growth of *L. monocytogenes* at 37°C. To further investigate whether the deletion of FepA influences colony morphology, we examined enlarged colonies utilizing a stereomicroscope. The colony morphology of *fepA* mutants was identical to that of EGD-e, displaying circular colonies with even edges and uniform sizes (Fig. 1B). These observations indicate that FepA is dispensable for the colonial appearance of *L. monocytogenes*.

### Loss of FepA increased sensitivity to a range of antimicrobial agents in *L. monocytogenes*

Following deletion of the *fepA* gene, the sensitivities of Δ*fepA* to cefotaxime (CTX), ampicillin (AMP), clindamycin (CLI), and tetracycline (TET) were all two-fold higher than that of wild-type strain EGD-e (Fig. 1D). Sensitivities to the other tested antibiotics, including previously reported fluoroquinolones, did not change clearly (data not shown). The most pronounced changes were observed in the MICs for disinfectants and dyes. The sensitivities of Δ*fepA* to disinfectants (BB; BC; chlorhexidine, Chlo) increased two-fold compared to EGD-e (Fig. 1D). Moreover, the MICs of Δ*fepA* to acriflavine (AF) and EtBr decrease by 4- and 16-fold, respectively (Fig. 1D). MIC values without or with 10 µg/mL reserpine are presented in Table S2. These findings indicate that FepA functions as a multidrug-resistant efflux pump, primarily bound up with resistance to disinfectants and dyes in *L. monocytogenes*.

### Deletion of FepA obviously weakened the growth of *L. monocytogenes* under antimicrobial pressure

**Fig 2:**
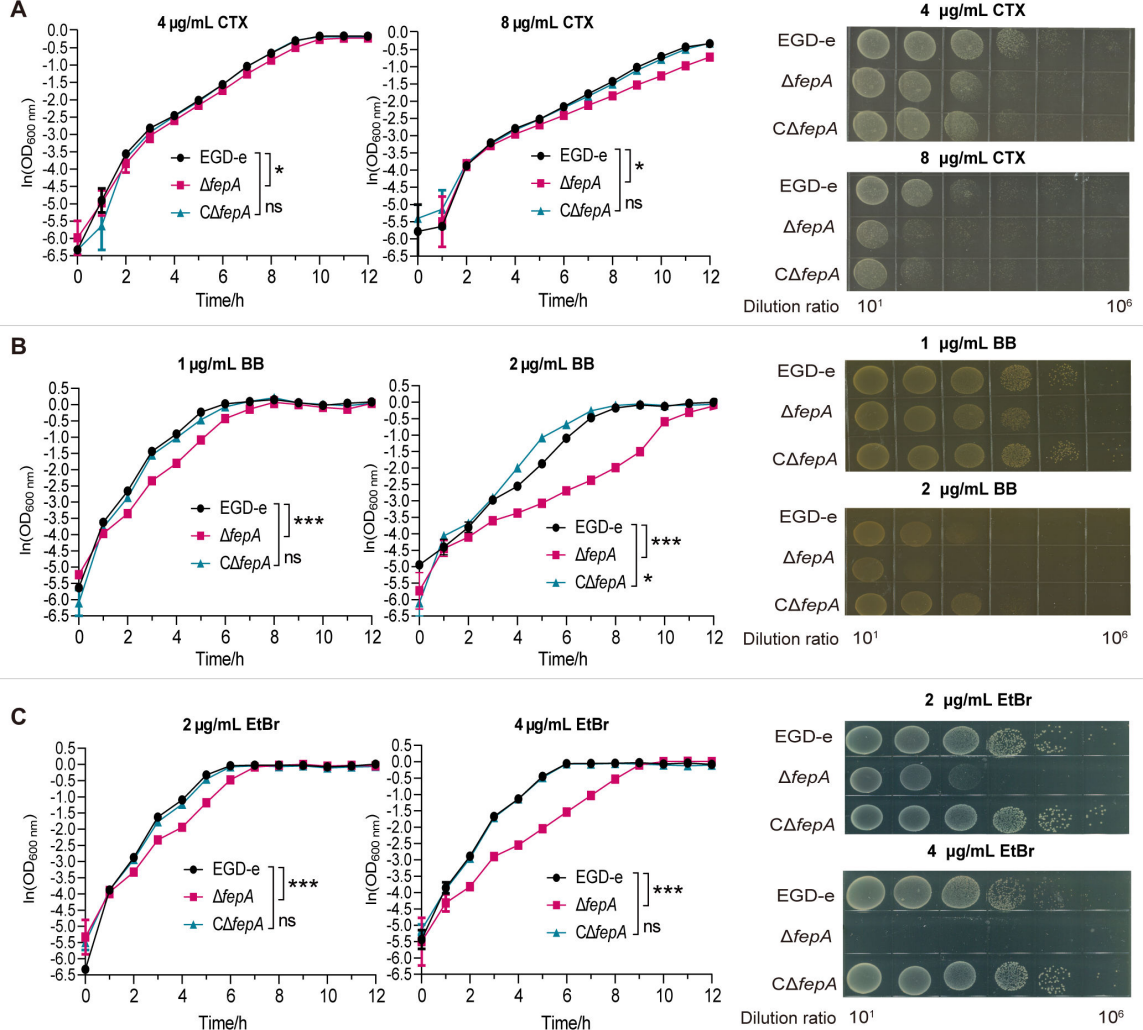
Deletion of FepA obviously weakened the growth of *L. monocytogenes* under the pressure of antimicrobial agents. Growth or survival of EGD-e and *fepA* mutants in medium containing different concentrations of CTX (4, 8 µg/mL, **A**), BB (1, 2 µg/mL, **B**), and EtBr (2, 4 µg/mL, **C**) in liquid BHI medium and BHI plates are presented. CTX, cefotaxime; BB, benzalkonium bromide; EtBr, ethidium bromide. Growth curves with logarithmic *Y*-axis are shown. Data are expressed as mean ± SD for three replicates. Also, growth rates in the exponential phase of three independent cultures per strain plus statistical analysis for comparison are calculated using GraphPad Prism 9.0. For the apparently restrained growth curves, the growth rate and lag time were simultaneously considered for statistical analysis. ns, no significance, *P < 0.05, ***P < 0.001. Representative images of three independent experiments on BHI plates are shown.

According to the MIC results, three antibacterial compounds, CTX, BB, and EtBr, were selected to assess survival under stress conditions. The survival capability of EGD-e, Δ*fepA*, and CΔ*fepA* in media containing 4–8 μg/mL CTX, 1–2 μg/mL BB, and 2–4 μg/mL EtBr is shown in Fig. 2A through C, respectively. At 8 µg/mL CTX, Δ*fepA* displayed increased sensitivity to cefotaxime in comparison to the wild-type strain EGD-e (*P* = 0.0466). (Fig. 2A). Under 2 µg/mL BB, Δ*fepA* exhibited a marked decrease in growth rate in liquid medium (*P* < 0.0001) and a 10-fold reduction in growth on solid medium compared to EGD-e (Fig. 2B). The most notable difference in survival was observed with EtBr. Under 2 µg/mL EtBr stress, the survival proficiency of Δ*fepA* was reduced by two orders of magnitude when compared to EGD-e on solid medium. The effect was even more pronounced at 4 µg/mL EtBr, where the survival ability of Δ*fepA* dropped by four orders of magnitude in comparison to EGD-e (Fig. 2C). Growth inhibition in liquid medium was also more severe for Δ*fepA* as the concentration of EtBr increased (*P* < 0.0001). Overall, these consequences demonstrated that FepA is essential in bacterial resistance to antimicrobial agents, particularly disinfectants and dyes.

### Absence of FepA did not influence the growth of *L. monocytogenes* at 30°C *in vitro*, but motility was considerably reduced

**Fig 3:**
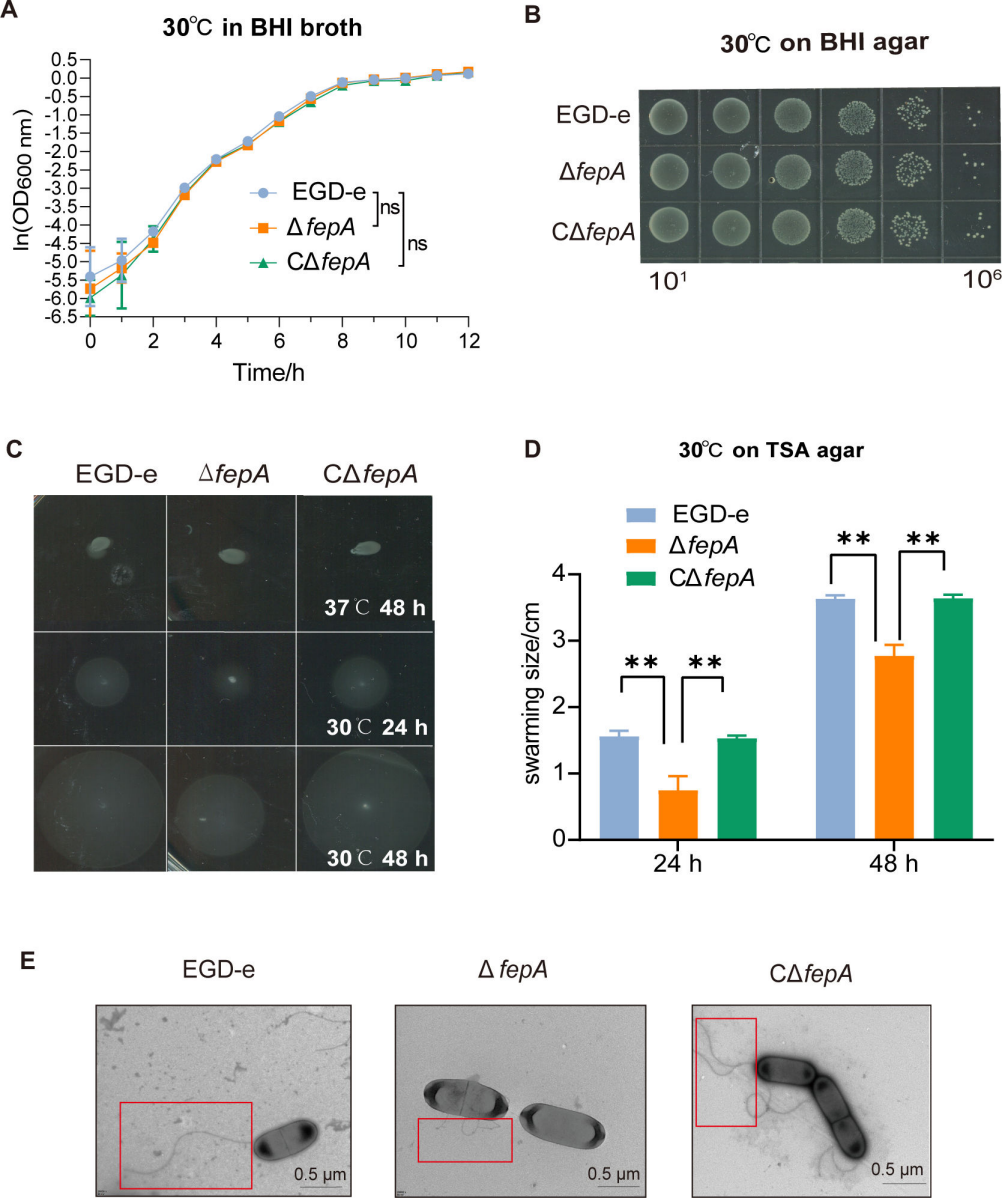
Absence of FepA did not affect the growth of *L. monocytogenes in vitro* at 30°C, but the motility was reduced considerably. (**A and B**) Growth curve and plate culture of wild-type EGD-e and *fepA* mutants at 30°C. (**A**) The growth curves are shown with a logarithmic *Y*-axis. Data are expressed as mean ± SD for three replicates. Growth rates in the exponential phase of three independent cultures per strain plus statistical analysis for comparison are calculated using GraphPad Prism 9.0. ns, no significance. (**B**) Representative images of three independent experiments are shown. (**C and D**) Motility assay and analysis of the EGD-e, *fepA* deletion, and complement strains at 30°C. Data are expressed as mean ± SD of three independent experiments. ns, no significance, **P < 0.01. (**E**) Effects of FepA on the flagellum morphology.

The growth curves and plate culture after 6 hours of growth in liquid culture proved that Δ*fepA* and CΔ*fepA* strains grew comparably to the wild-type strain at 30°C (*P* = 0.3750 and *P* = 0.5968, respectively) (Fig. 3A and B), indicating that the absence of FepA does not influence the growth of *L. monocytogenes* at the temperature. However, when cultured at 30°C for 24 and 48 hours, the swimming diameters of Δ*fepA* were pronouncedly smaller than those of EGD-e and CΔ*fepA* (Fig. 3C), as confirmed by statistical analysis (all at the *P* < 0.01 level) (Fig. 3D). The outcomes imply that the deletion of FepA reduces the motility of *L. monocytogenes in vitro*. Transmission electron microscopy of fresh single bacterial colonies cultured at 30°C for 18 hours revealed that the flagellum length of Δ*fepA* was distinctly shortened compared with that of EGD-e and CΔ*fepA* (Fig. 3E). This suggests that FepA affects the motility of *L. monocytogenes* by influencing flagellum synthesis.

### Decreased transcription levels of flagellar genes and expression levels of flagellar biosynthesis regulatory proteins such as FlhF were observed in Δ*fepA*

**Fig 4:**
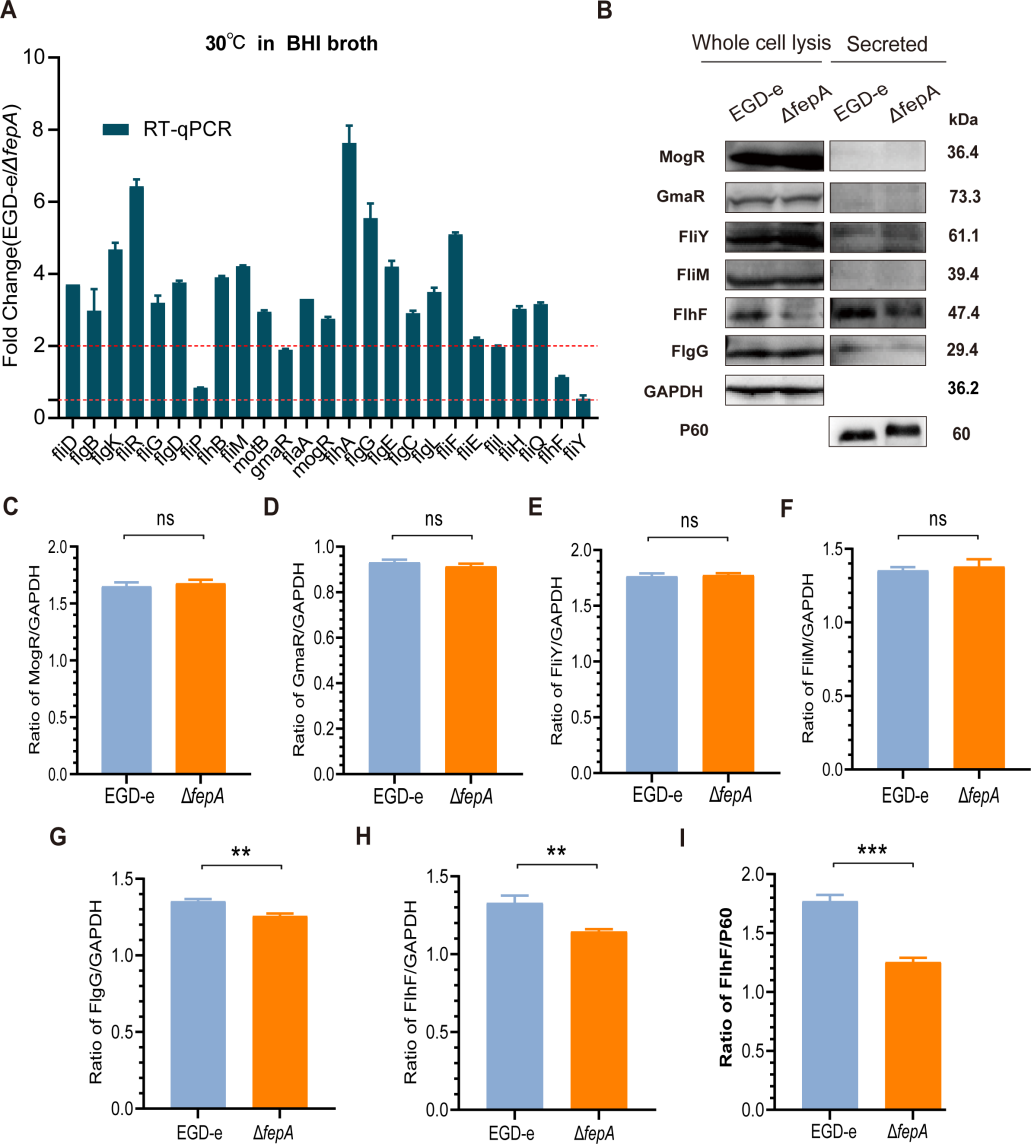
Declined transcription levels of flagellar genes and expression levels of flagellar proteins were proved in Δ*fepA*. Transcriptional change of flagellum-associated genes (**A**) and representative image for expression changes of partial flagellum-associated proteins (**B**) after deletion of *fepA* were presented. (**A**) Data are expressed as mean ± SD for three replicates. Values exceeding two point to an increase, whereas values under 0.5 suggest a decrease. (**C–I**) The WB results of repeated experiments were analyzed by using ImageJ software for quantitation. Data are expressed as mean ± SD for three replicates. ns, no significance, **P < 0.01, ***P < 0.001. Second representative image of WB experiments and quantitative ImageJ analysis of expression levels are presented in Fig. S1.

After deletion of FepA, the transcription levels of most flagellar genes decreased significantly (Fig. 4A), including regulatory gene (*mogR*), genes encoding flagellar filament proteins (*flaA and fliD*), hook and junction proteins (*flgD*, *flgE*, *flgK,* and *flgL*), rod proteins (*flgB*, *flgC*, *fliE,* and *flgG*), and basal body and other flagellar synthesis-related proteins (*motB*, *fliR*, *fliM*, *fliG*, *flhA*, *flhB*, *fliF*, *fliH,* and *fliQ*). There was no notable difference in the transcription levels of *flhF, fliY,* and *fliP*. These results suggest that FepA promotes flagellum formation in *L. monocytogenes* by influencing the transcription of a broad array of flagellum-related genes. To further analyze the effects of FepA on flagellum formation, we conducted WB analyses on whole bacterial and secreted protein from EGD-e and Δ*fepA* strains (Fig. 4B). The expression levels of some flagellum-related proteins were examined, and gray-scale analysis was performed. In comparison to the wild-type strain EGD-e, the expression levels of MogR, GmaR, FliY, and FliM were all observed at the *P* > 0.05 level (Fig. 4C through F). However, the expression levels of FlgG (*P* = 0.0014) and FlhF (*P* = 0.0028), particularly FlhF, were noticeably decreased in Δ*fepA* (Fig. 4G and H). A substantial decrease in secreted FlhF protein (*P* = 0.001) was observed in Δ*fepA* relative to the EGD-e strain, but not in FlgG (Fig. 4I). FlhF is annotated as a flagellar biosynthetic protein. Therefore, the results indicated that FepA likely affected bacterial flagellum formation by influencing flagellar biosynthetic protein FlhF and the flagellar rod protein FlgG, thus reducing the motility of *L. monocytogenes*. Due to the limited number of available antibodies against flagellar proteins, additional research is required to ascertain whether FepA affects the expression of other flagellar proteins.

### FepA deletion did not affect bacterial cell-to-cell spread efficiency but impaired proliferation in RAW264.7 macrophages and invasion of Caco-2 cells

**Fig 5:**
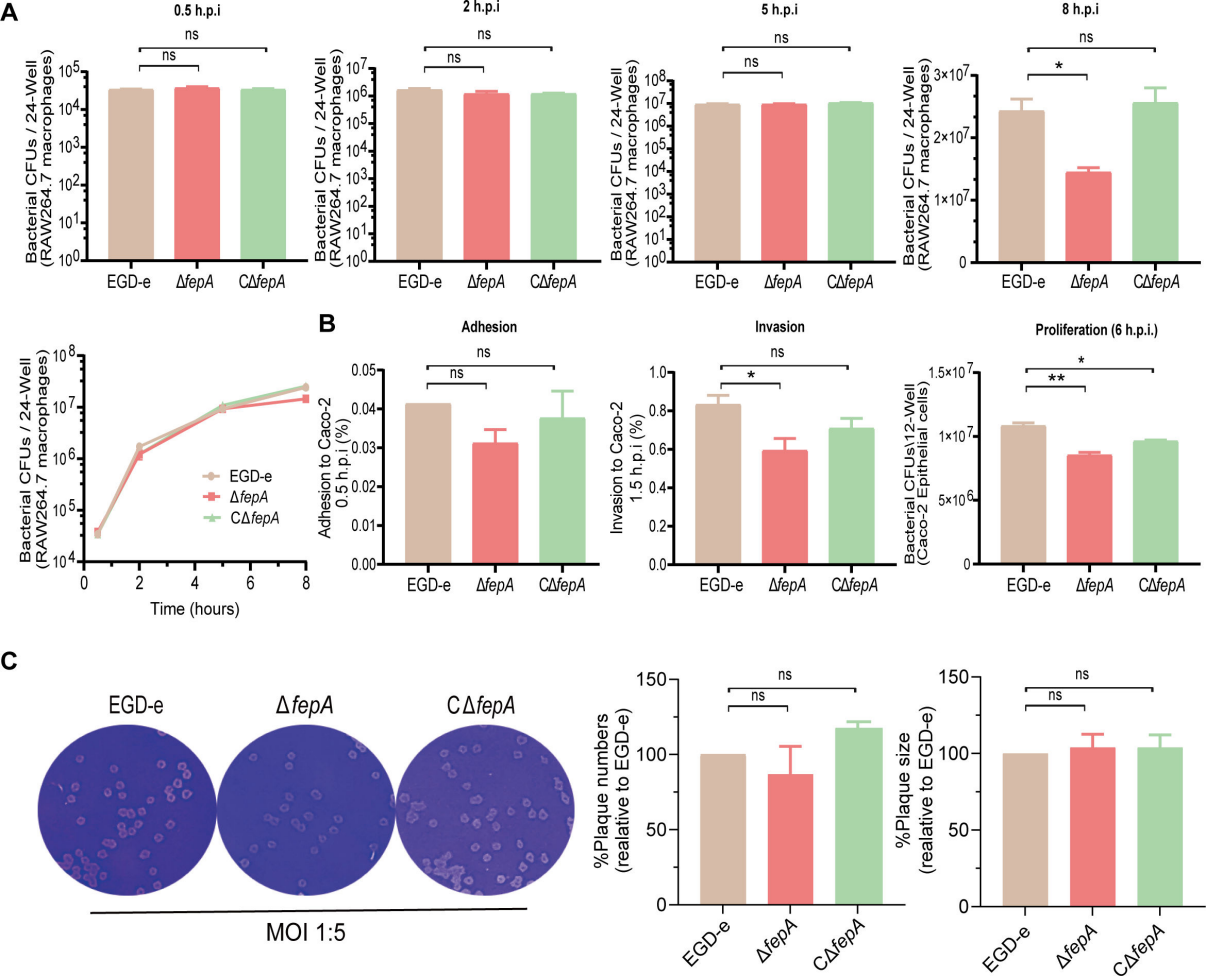
FepA deletion did not affect the efficiency of bacterial cell-to-cell spread but impaired the proliferation of bacteria in RAW264.7 macrophages and the invasion ability of bacteria to Caco-2 cells. (**A**) Intracellular growth of wild-type *L. monocytogenes* and *fepA* mutants in RAW264.7 macrophages. The number of recovered bacteria able to invade cells and survive is expressed as mean ± SD of three replicates for each strain. ns, no significance, *P < 0.05. (**B**) Adhesion and invasion of *L. monocytogenes* in human intestinal epithelial cells, Caco-2. The number of bacteria able to invade cells and survive is expressed as mean ± SD of the recovery rate for each strain (three replicates). ns, no significance, *P < 0.05, **P < 0.01. (**C**) The plaque assay was performed using L929 fibroblasts. The plaque size and numbers of the mutant strains were indicated as a percentage of those formed by the wild-type strain. Data are expressed as mean ± SD of randomly selected plaques (100 plaques for size comparison) for each strain. ns, no significance. Representative plaque formation images of three independent experiments are shown.

The deletion of FepA did not impact the number or size of plaques formed upon infection of L929 fibroblast monolayers by *L. monocytogenes* (*P* = 0.0535), indicating that the absence of FepA does not affect the bacteria’s ability to spread cell-to-cell (Fig. 5C). However, regarding intracellular infection in the RAW264.7 macrophages, there were no notable differences in bacterial counts among EGD-e and *fepA* mutants during the initial 0.5, 2, and 5 hours of infection (all at the *P* > 0.05 level) (Fig. 5A). Despite this, a steady decrease in bacterial proliferation was observed up to 8 hours post-infection (*P* = 0.0203) (Fig. 5A), suggesting that deletion of FepA does impair the proliferation of *L. monocytogenes* within RAW264.7 cells. Furthermore, the invasion rate (*P* = 0.0481) and proliferation (*P* = 0.0084) of Δ*fepA* in Caco-2 cells were notably diminished compared to those observed in the wild-type EGD-e strain (Fig. 5B), indicating that FepA is crucial for the invasion ability of *L. monocytogenes*.

### The transcription level or expression level of certain virulence genes and proteins decreased substantially after FepA deletion

**Fig 6:**
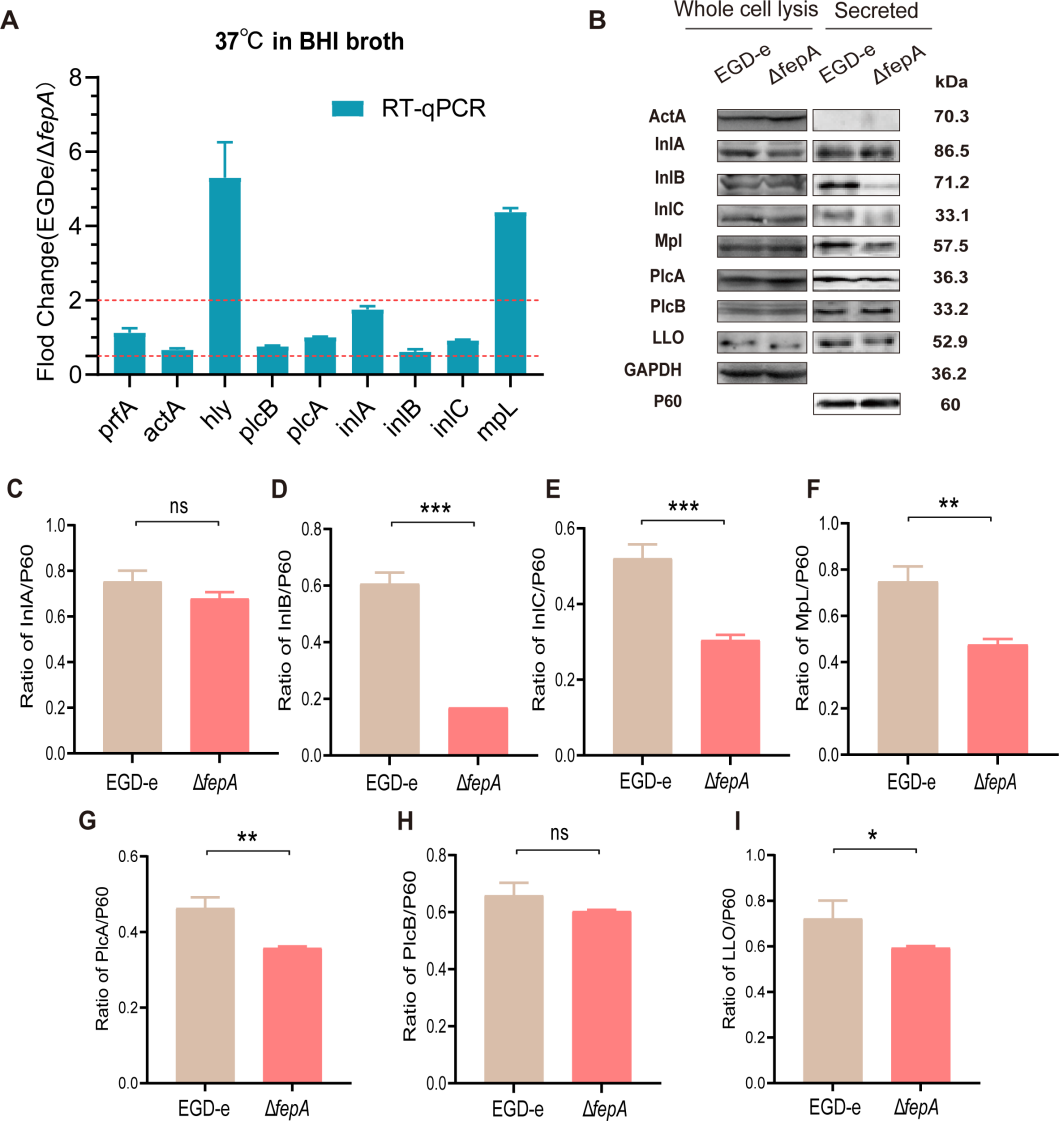
The transcription level or expression level of some virulence genes or proteins decreased significantly after FepA deletion. Transcriptional change of virulence genes (**A**) and representative image for expression changes of partial virulence proteins (**B**) after deletion of FepA were presented. (**A**) Data are expressed as mean ± SD for three replicates. Values exceeding two point to an increase, whereas values under 0.5 suggest a decrease. (**C–**I) The WB results of repeated experiments were analyzed by using ImageJ software for quantitation. Data are expressed as mean ± SD for three replicates. ns, no significance, *P < 0.05, **P < 0.01, ***P < 0.001. Second representative image of WB experiments and quantitative ImageJ analysis of expression levels are presented in Fig. S2.

Previous results indicated that the absence of FepA reduces the virulence of *L. monocytogenes*. To explore the underlying mechanism, we examined the transcription levels of nine virulence genes in EGD-e and Δ*fepA* strains. As shown in Fig. 6A, no statistically significant differences were detected in the transcriptional levels of *prfA*, *actA*, *plcB*, *plcA*, *inlA*, *inlB*, and *inlC* between Δ*fepA* and EGD-e. However, the transcription levels of *hly* and *mpl* were reduced in the Δ*fepA* strain. Further analysis using WB and gray-scale analysis revealed that there was a pronounced reduction in the levels of secreted virulence proteins InlB, InlC, Mpl, PlcA, and LLO in Δ*fepA* compared to the EGD-e strain (all at the *P* < 0.05 level), whereas the levels of InlA and PlcB were not affected significantly (Fig. 6B through I). Collectively, these findings suggest that FepA likely promotes the secretion of bacterial virulence proteins (such as InlB, InlC, Mpl, PlcA, and LLO), thereby contributing to the invasion and proliferation capabilities of *L. monocytogenes*.

## DISCUSSION

Efflux pumps apparently play a very important role in resistance to various adverse external environments for *L. monocytogenes* (42). According to previous reports, efflux pumps participate in the pumping of various antibiotics, disinfectants, dyes, heavy metals, and other toxic compounds in *L. monocytogenes* (43). Previously, there have been a few reports on efflux pumps related to the resistance of disinfectants and dyes, such as MdrL, which is involved in exporting macrolide antibiotics, cefotaxime, heavy metals, EtBr, and the disinfectant BC (15, 44). The efflux pump Lde conferring fluoroquinolone resistance in *L. monocytogenes* can mediate the export of ciprofloxacin, norfloxacin, acriflavine, EtBr, etc. (38, 45). FepA is the only MATE family efflux pump currently reported in *L. monocytogenes*, conferring resistance to several antibiotics, disinfectants, and dyes. The initial finding of FepA revealed its connection not only to resistance against fluoroquinolone antibiotics but also to tolerance of quaternary ammonium compounds (QACs), due to a point mutation in the upstream regulatory gene *fepR* causing overexpression of FepA (30). Further, the study on cross-resistance between commercial QACs and antibiotics in *L. monocytogenes* from food processing environments discovered that the role of *fepR* mutations is significant in the decline of antibiotic susceptibility after low-level QACs adaptation (46).

A more systematic analysis of sequences highlighted the correlation between *fepR* mutations and tolerance to QACs. Through comparative genomics, core genome single nucleotide polymorphism (SNP) analysis discovered numerous mutations in the transcriptional regulator *fepR* in 94% of disinfectant-adapted strains, while mutations in other genes occurred less frequently (47). Previous research further revealed that low-dose BC induces mutations in the *fepR* gene of most *L. monocytogenes* strains, thereby raising their BC tolerance concentration. Nonetheless, it did not notably enhance the survival of these resistant strains at the advised disinfectant levels in food processing settings (48). These outcomes demonstrated that the recommended concentration of BC used for disinfection in food processing environments has a similar impact on killing *L. monocytogenes* no matter if the strains can withstand low concentrations of BC, pointing out that while BC-resistant *L. monocytogenes* strains may not be a major concern, continuous observation is still needed (48). Moreover, various mechanisms by which *L. monocytogenes* adapts to endure two significant QACs, BC and cetyltrimethylammonium bromide (CTAB), were discussed, including *fepR* mutations and compensatory overproduction of other efflux systems. These findings provide clarity on the persistent presence and proliferation of *L. monocytogenes* in the food industry (33).

Bacterial survival in food processing environments is promoted by resistance to disinfectants and dyes, thereby raising the risk of infection (42, 49). Consistently, the export of disinfectants and dyes demonstrated the importance of FepA for the endurance of *L. monocytogenes* in food processing settings.

*Listeria monocytogenes* are usually sensitive to a variety of antimicrobial agents, but they have inherent or natural resistance to certain antimicrobial agents, including first-generation quinolones, fosfomycin, and cephalosporins of the third generation (50). Since cephalosporins are the most commonly used antibiotics for the treatment of sepsis caused by unknown reasons, inherent resistance to cephalosporins remarkably reduces the cure rate of *L. monocytogenes* infection (51). The mechanisms of inherent resistance to cephalosporins in *L. monocytogenes* are quite complex, including penicillin-binding proteins, multiple efflux pumps, like MdrL, AnrAB, and VirAB, two-component signal transduction systems, like CesRK, LisRK, and some other regulators (15, 18, 51–54). Our study demonstrated that FepA also contributes to inherent resistance to cefotaxime. However, FepA deletion did not affect fluoroquinolone susceptibility, possibly due to the compensatory action of other efflux pumps such as Lde (38).

Motility is one of the important factors that allow bacteria to settle on different surfaces (55). In the past two decades, studies have demonstrated the connection among different efflux pumps and bacterial motility. Inactivation of a previously undescribed efflux pump, MexGHI-OpmD, in *Pseudomonas aeruginosa*, which provides resistance to vanadium, leads to changes in many important biological functions, including a decrease in swarming ability. This is particularly true for the *mexI* mutant, which does not swarm at all (56). It is thought that the MexGHI-OpmD pump likely participates in AHL homeostasis, playing a role in the quorum sensing network, thereby regulating the swarming motility (56). In *Salmonella*, the AcrD efflux pump is linked to impaired motility, possibly due to metabolic disruptions (57). In *Stenotrophomonas maltophilia,* the SmeYZ efflux pump affects swimming and flagellum formation apart from contributing resistance to aminoglycosides and trimethoprim-sulfamethoxazole, showcasing its pleiotropic effects (58). However, no direct association between the efflux pump and motility of *L. monocytogenes* has been documented. Efflux pumps enable bacteria to withstand harmful environmental substances like antibiotics and antiseptics (42), while motility aids them in finding better growth conditions (55). For example, findings suggest that *L. monocytogenes* strains missing efflux pump genes such as *fepA* or *sugE1/2* can still gain antimicrobial tolerance by enhancing the expression of other efflux mechanisms (33). Gene mutations affecting flagellar motility have been associated with altered CTAB tolerance in *L. monocytogenes* (59). These findings propose a connection between the motility of *L. monocytogenes* and its resistance to antimicrobials. Furthermore, efflux pumps and motility are both connected to the formation of biofilms (16, 26, 60). Efflux pumps might also control the levels of metabolites or signaling molecules in bacteria, possibly via quorum sensing, which indirectly influences the expression of flagellar genes and thereby affects motility (56, 61). Comprehending the mechanisms of *Listeria*’s motility and efflux pump regulation is crucial for addressing antimicrobial resistance in this pathogen. Our study is the first to show that the FepA efflux pump in *L. monocytogenes* promotes bacterial motility by influencing flagellum synthesis. The transcription and expression levels of flagellum-related genes and proteins suggest that FepA is heavily involved in these processes (Fig. 4). Further studies are needed to determine if FepA affects motility through quorum sensing or other metabolic functions. The bacterium *L. monocytogenes* forms flagella as a means of movement at or below 30°C when outside a host but ceases flagellar expression at 37°C inside a human or animal host to escape immune responses triggered by flagella (62). Consequently, our attention is limited to the formation of flagella *in vitro*.

Efflux pumps also have roles in bacterial virulence (63, 64). They contribute to the production or secretion of virulence factors and the exporting of biofilm structural elements or quorum sensing signals, which control the expression of virulence genes (60, 63–65). The results of cellular experiments showed that FepA deletion did not affect the adhesion and cell-to-cell spread of *L. monocytogenes* but could decrease the ability of invasion to epithelial cells and replication within macrophages (Fig. 5). The deletion of FepA remarkably decreased the expression of internalin-related proteins, particularly InlB and InlC, impairing the secretion of these proteins without affecting their transcription (Fig. 6). This indicates that FepA facilitates invasion and proliferation by promoting internalin protein secretion. Internalin proteins InlA and InlB engage with specific cell surface receptors to promote *L. monocytogenes* entry into non-phagocytic cells (66). The reduced transcription and expression levels of the primary virulence factors LLO and Mpl in the *fepA*-deficient strain likely contribute to the decreased invasion and proliferation capabilities in epithelial cells as well. The secretion of virulence factors in *L. monocytogenes* mainly depends on the Sec secretion system, particularly SecA2 (67, 68). The ESX-1 secretion system, known from *Mycobacterium tuberculosis,* also plays a role in *L. monocytogenes* pathogenicity (69, 70). Our results suggest that FepA promotes virulence expression by aiding in the secretion of proteins such as InlB, InlC, Mpl, PlcA, and LLO. Further studies are needed to explore FepA’s relationship with the SecA2 or ESX-1 secretion systems.

### Conclusions

This study comprehensively investigated the role of the MATE efflux pump FepA in *L. monocytogenes*. It is found that FepA mediates resistance to various drugs and compounds, and FepA deletion affects flagellum formation by influencing the flagellar biosynthetic protein FlhF and the flagellar rod protein FlgG, reducing bacterial motility. Additionally, the absence of FepA impairs bacterial invasion and intracellular proliferation due to decreased secretion of virulence proteins such as InlB, InlC, Mpl, PlcA, and LLO. These discoveries offer further understanding about the processes of adapting to the environment and virulence in *L. monocytogenes* and offer a theoretical basis for improving infection treatment outcomes.

## SUPPLEMENTAL MATERIAL

Supplemental material aem.00462-25-s0001.docx Tables S1 and S2; Figures S1 and S2.

ASM does not own the copyrights to Supplemental Material that may be linked to, or accessed through, an article. The authors have granted ASM a non-exclusive, world-wide license to publish the Supplemental Material files. Please contact the corresponding author directly for reuse.
