# Supplementary material for: Beyond antimicrobial resistance: MATE-type efflux pump FepA contributes to flagellum formation and virulence in Listeria monocytogenes
Source: Appl Environ Microbiol. 2025 Jun 10;91(7):e00462-25. doi: 10.1128/aem.00462-25 (PMC12285229; doi:10.1128/aem.00462-25)
Supplement: Supplemental material — Tables S1 and S2; Figures S1 and S2. [file aem.00462-25-s0001.docx]

**Table S1**. Primers used in this study

| Primers | Sequences (5′→3′) |
| --- | --- |
| Δ*fepA*-a-*Bam*HI | CGCGGATCC acttcgagatagtgacttatccagaccag |
| Δ*fepA*-b | aacgcccgggataccagacattaacaacattcccactaaggaagg |
| Δ*fepA*-c | atgttgttaatgtctggtatcccgggcgtttggcta |
| Δ*fepA*-d-*Pst*I | AAAACTGCAGaacctaaagcgttcgcccttacatAGA |
| CΔ*fepA*-a-*Sac*I | agcgagctcttctttcgccattttaaacatccc |
| CΔ*fepA*-b | catattttttgccattttcacattcgctcaactcct |
| CΔ*fepA*-c | tgagcgaatgtgaaaatggcaaaaaatatggaaattttagaaac |
| CΔ*fepA*-d-*Bam*HI | CGCGGATCCttatttaaataaaatatgttttttcttcatatagaatacaatgg |
| *gmaR*-RT-fwd | AGCAAGTTCCATCAACCAAAAG |
| *gmaR*-RT-rev | GTTGAGTTGTCATCGAAAGTAAGC |
| *mogR*-RT-fwd | TCTGAAATGCTCAGCCTAAA |
| *mogR*-RT-rev | TCGGAATATCTTCTACTTGGA |
| *motB*-RT-fwd | AAGCGTCGCAAGAAACCG |
| *motB*-RT-rev | GAGGCAAACAGAACGATAAATAGAG |
| *fliR*-RT-fwd | GAACATTCCGAACAGTGTCAAGG |
| *fliR*-RT-rev | AGCGTCGTAATACCAGAAACATCCA |
| *fliP*-RT-fwd | TACGGTTCTTTCTTTGTCTGCA |
| *fliP*-RT-rev | CCAAGACCCTGCCTCGTTA |
| *fliY*-RT-fwd | AATGAACGACCTTGCCTCGT |
| *fliY*-RT-rev | GCCATGATTAACGCGGCATT |
| *fliG*-RT-fwd | CGCTTATTATTTGGAGCCTTGA |
| *fliG*-RT-rev | CGCCGTCCATTTCTTTCATT |
| *fliH*-RT-fwd | ACGTCACCACCCACCCAGAA |
| *fliH*-RT-rev | CGCCGTAATCCAGTCGCATAT |
| *fliD*-RT-fwd | AAGGTGATTCGGCTGTTCTCG |
| *fliD*-RT-rev | CACGCCTGGGATGTAGTTGG |
| *fliE*-RT-fwd | ATGCAGCCAGCGTCTTACC |
| *fliE*-RT-rev | CTATCCAGCATTTGCGTGAAG |
| *fliI*-RT-fwd | CGTAACGCCAAAGCAGACAT |
| *fliI*-RT-rev | TCCGAGGTAGCAGCAACAAT |
| *fliF*-RT-fwd | TCACCATCATGCCTATCCAGTT |
| *fliF*-RT-rev | GATGACGGCGAAGAGTAAACC |
| *flgB*-RT-fwd | TTTGCGAGCATCCGGTCAG |
| *flgB*-RT-rev | GTTTCGTCTATGTCGGTTCCAG |
| *flgC*-RT-fwd | ACAAGCGGCTCTGCGTTAA |
| *flgC*-RT-rev | GGCGTGATTTCGGATAGGAC |
| *flgD*-RT-fwd | ATTAGTAGTTTATCAGGAGCGAGTCAG |
| *flgD*-RT-rev | AGTCATCTTTGCCAAGGGTTT |
| *flgE*-RT-fwd | GCCAATGCCAACACGACA |
| *flgE*-RT-rev | CGAACCGGAACCGAAACT |
| *flgG*-RT-fwd | CCAGTTTCATTCCGATCAGTTT |
| *flgG*-RT-rev | TTGAATCGCTTCTCCAGTCG |
| *flgK*-RT-fwd | TTACGGTGACGGTGGTCTACTTG |
| *flgK*-RT-rev | GCGGATTGTTTGGCGATTT |
| *flgL*-RT-fwd | TAACTCAGAAGACGATGGCAGAT |
| *flgL*-RT-rev | TTCGGTTGTCCCGTTATAGATG |
| *flhA*-RT-fwd | CAGCGGAGGTAGCAGCAAGA |
| *flhA*-RT-rev | GGTTTCCATATTCAAGTAAGCACGT |
| *flhB*-RT-fwd | GAGGTCGTGAAAGCGTTATTGT |
| *flhB*-RT-rev | CGGTGTAACTAATGAGCGTATCTAA |
| *flhF*-RT-fwd | GGGGAACTCCAAGCGAGAAA |
| *flhF*-RT-rev | GCTTTGTGACACGTTCGTCC |
| *fliM*-RT-fwd | CACTCAAAGGCTATGTCACCG |
| *fliM*-RT-rev | TGCTCTGTCCGCCCTGTAT |
| *flaA*-RT-fwd | CTTGATGACGCTGCTGGTCT |
| *flaA*-RT-rev | AGCTGAATCCGCTGTTTGTA |
| *fliQ*-RT-fwd | TGGTAGTGATTGTGGTTGCGATTT |
| *fliQ*-RT-rev | ATCCACGGTCCAAGAATAAAGA |
| *prfA*-RT-fwd | AGCTTGGCTCTATTTGCGGT |
| *prfA*-RT-rev | CGATGCCACTTGAATATCCTAACT |
| *actA*-RT-fwd | CAGCAGATGAGTCTTCACCACA |
| *actA*-RT-rev | CCATTTCCCCGCATCTTTTA |
| *hly*-RT-fwd | TCACATCGTCCATCTATTTGCC |
| *hly*-RT-rev | ATTACCGTTCTCCACCATTCC |
| *plcB*-RT-fwd | CGCCCTTTTCGCATTTTC |
| *plcB*-RT-rev | ATCATACCCTCCAGGCTACCA |
| *plcA*-RT-fwd | CGAGCAAAACAGCAACGATAG |
| *plcA*-RT-rev | CGTGTCAGTTCTGGGAGTAGTGTAA |
| *inlA*-RT-fwd | TAGCGATGGCGGTAGTTACACAGA |
| *inlA*-RT-rev | TTAAGTGGCTGCGTCACGGTTC |
| *inlB*-RT-fwd | GCAGTCAACTTAACCCGCTATGTCA |
| *inlB*-RT-rev | GTGCGAGGCTAGAGTTCCTTGTT |
| *inlC*-RT-fwd | GAGTATTCAACGACCAACGCCTAT |
| *inlC*-RT-rev | GGTCTGTAACACTTTGCTTCCCTA |
| *mpl*-RT-fwd | CAGCAAGGACAGCTTAGGATTAC |
| *mpl*-RT-rev | CTTTCACTGGGTTTCCGACATA |

**Table S2.** MIC values to the antimicrobial agents of wild-type EGD-e and *fepA* mutants with or without reserpine

| ***L. monocytogenes*** | **MIC (μg/mL)** | | | | | | | | | | | | | | | | | |
| --- | --- | --- | --- | --- | --- | --- | --- | --- | --- | --- | --- | --- | --- | --- | --- | --- | --- | --- |
|  | **Cefotaxime** | | **Clindamycin** | | **Ampicillin** | | **Tetracycline** | | **Acriflavine** | | **Ethidium bromide** | | **Benzalkonium bromide** | | **Benzalkonium chloride** | | **chlorhexidine** | |
|  | **-** | **+** | **-** | **+** | **-** | **+** | **-** | **+** | **-** | **+** | **-** | **+** | **-** | **+** | **-** | **+** | **-** | **+** |
| EGD-e | 8 | 8 | 0.5 | 0.5 | 0.25-0.5 | 0.25-0.5 | 0.125-0.25 | 0.125-0.25 | 16 | 16 | 32 | 16 | 4 | 2 | 2 | 1 | 0.5 | 0.25 |
| △*fepA* | 4 | 4 | 1 | 1 | 0.25 | 0.25 | 0.25 | 0.25 | 4 | 4 | 2-4 | 2 | 2 | 1 | 1 | 0.5 | 0.125-0.25 | 0.125 |
| C△*fepA* | 8 | 8 | 0.5-1 | 0.5 | 0.25-0.5 | 0.25-0.5 | 0.125-0.25 | 0.125 | 8-16 | 8 | 32 | 16 | 4-8 | 2-4 | 2-4 | 1 | 0.5 | 0.25 |

-, without reserpine; +, with 10 μg/mL of reserpine.


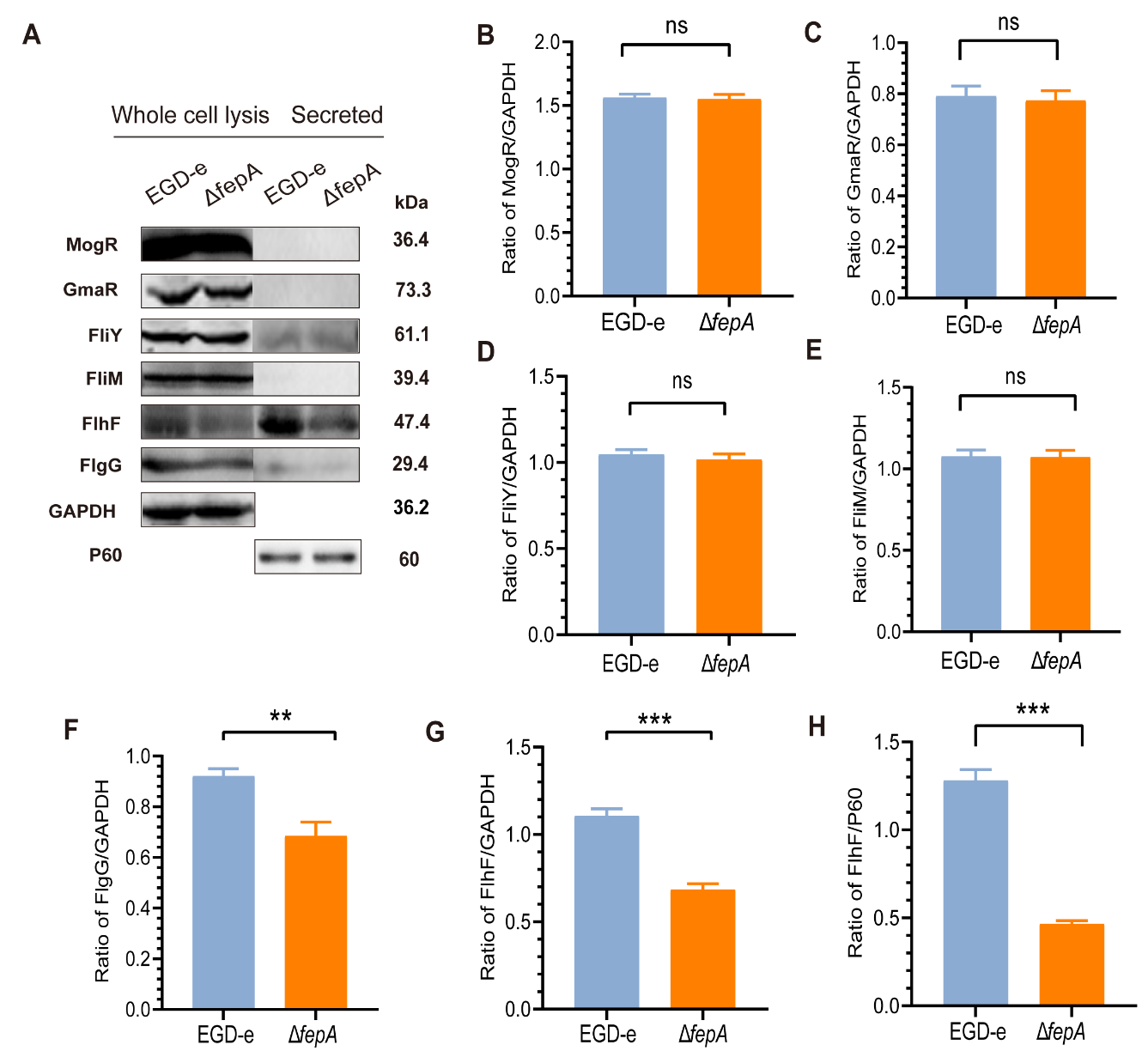


**Figure S1. Second representative image of WB experiments and quantitative Image J analysis of expression levels for flagellar proteins.** Image for expression changes of partial flagella-associated proteins (A) after deletion of *fepA* were presented. (B-H) The WB results were analyzed by using Image J software for quantitation. Data are expressed as mean ± SD for three replicates. ns, no significance, ***P* < 0.01, ****P* < 0.001.

**
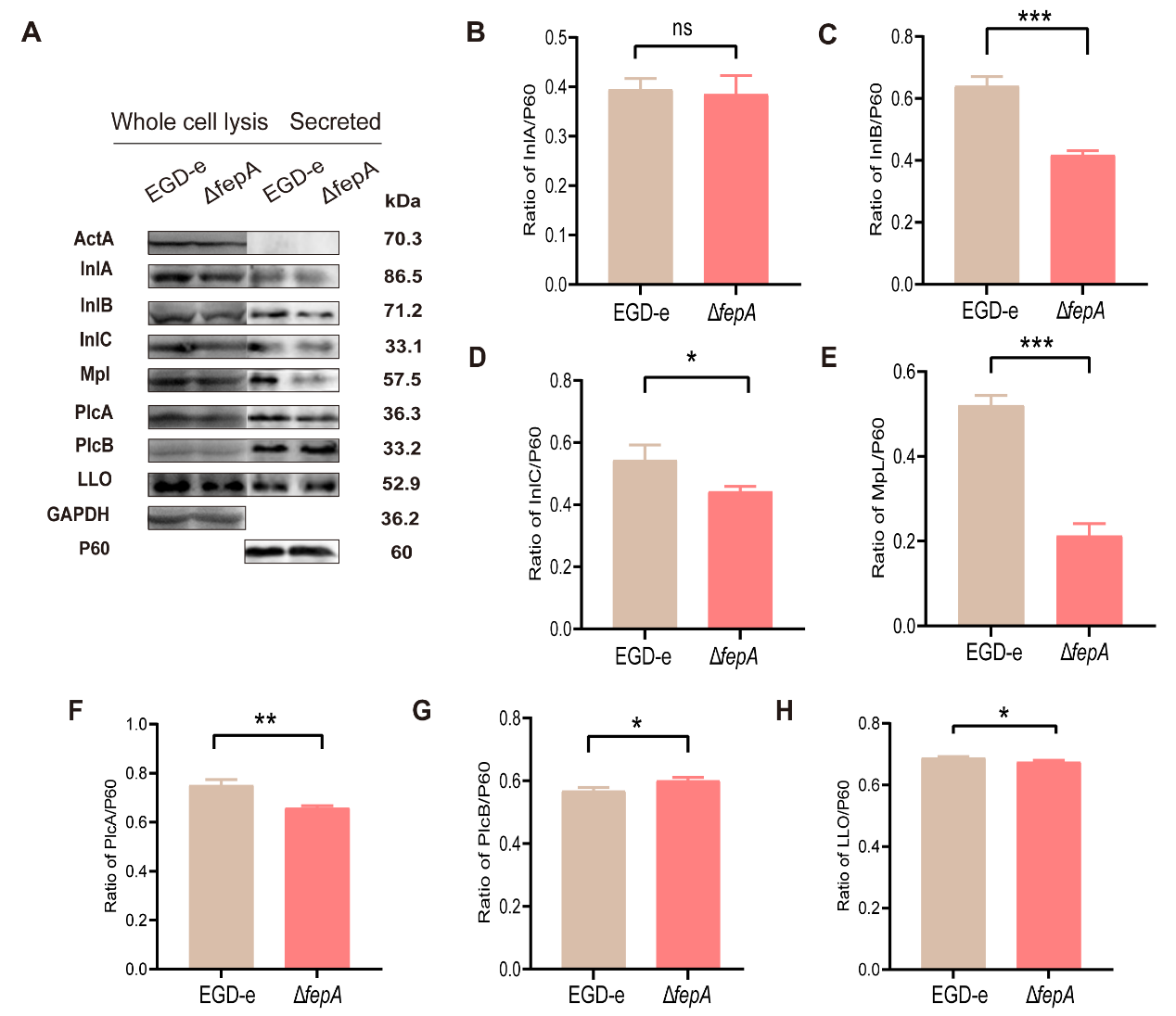
**

**Figure S2. Second representative image of WB experiments and quantitative Image J analysis of expression levels for virulence proteins.** Image for expression changes of partial virulence-associated proteins (A) after deletion of *fepA* were presented. (B-H) The WB results were analyzed by using Image J software for quantitation. Data are expressed as mean ± SD for three replicates. ns, no significance, **P* < 0.05, ***P* < 0.01, ****P* < 0.001.
